# Selectivity of biopolymer membranes using HepG2 cells

**DOI:** 10.1093/rb/rbu018

**Published:** 2015-02-09

**Authors:** Dongyuan Lü, Yuxin Gao, Chunhua Luo, Shouqian Lü, Qian Wang, Xianghong Xu, Shujin Sun, Chengzhi Wang, Mian Long

**Affiliations:** ^1^Center of Biomechanics and Bioengineering and Key Laboratory of Microgravity (National Microgravity Laboratory), Institute of Mechanics, Chinese Academy of Sciences, Beijing 100190, China; ^2^State Key Laboratory of Nonlinear Mechanics, Institute of Mechanics, Chinese Academy of Sciences, Beijing 100190, China

**Keywords:** HepG2, biocompatible membrane, bioartificial liver

## Abstract

Bioartificial liver (BAL) system has emerged as an alternative treatment to bridge acute liver failure to either liver transplantation or liver regeneration. One of the main reasons that the efficacy of the current BAL systems was not convincing in clinical trials is attributed to the lack of friendly interface between the membrane and the hepatocytes in liver bioreactor, the core unit of BAL system. Here, we systematically compared the biological responses of hepatosarcoma HepG2 cells seeded on eight, commercially available biocompatible membranes made of acetyl cellulose–nitrocellulose mixed cellulose (CA–NC), acetyl cellulose (CA), nylon (JN), polypropylene (PP), nitrocellulose (NC), polyvinylidene fluoride (PVDF), polycarbonate (PC) and polytetrafluoroethylene (PTFE). Physicochemical analysis and mechanical tests indicated that CA, JN and PP membranes yield high adhesivity and reasonable compressive and/or tensile features with friendly surface topography for cell seeding. Cells prefer to adhere on CA, JN, PP or PTFE membranes with high proliferation rate in spheriod-like shape. Actin, albumin and cytokeratin 18 expressions are favorable for cells on CA or PP membrane, whereas protein filtration is consistent among all the eight membranes. These results further the understandings of cell growth, morphology and spreading, as well as protein filtration on distinct membranes in designing a liver bioreactor.

## INTRODUCTION

Liver is a vital organ thought to be responsible for up to 500 distinct functions usually in combination with other systems and organs, including detoxification, synthesis, storage and digestion [1]. One of the clinical strategies for treating acute liver failure is to maintain essential liver functions and to extend the survival duration of patients in critical phase until donor livers are available and liver transplantation can be achieved [2]. In the past decades, attempts have been made to develop various bioartificial liver (BAL) systems capable of providing transient support to patients with liver failure [3]. Liver bioreactor, a core unit of BAL system, usually consists of layered supporting membranes for hepatocyte immobilization and of flowing channels for blood perfusion and medium exchange. Thus, hepatocyte–membrane interactions are important in constructing the friendly interface in designing a liver bioreactor.

Artificial membranes are usually made of synthetic biopolymer used for separation purposes in biochemical engineering. They are chemically, thermally and mechanically stable, and biologically inert, and often categorized as dense, porous and asymmetric membranes in distinct structures/morphologies. These porous membranes are widely applicable in the microfiltration, ultrafiltration and dialysis based on the given pore size and/or the cut-off molecular weight for isolating specific cells or biomacromolecules. Most commonly used membranes include cellulose acetate (CA), nitrocellulose (CN), polytetrafluoroethylene (PTFE), polyamide (nylon, JN), polypropylene (PP) and polyvinylidene fluoride (PVDF) membranes. Nowadays, these bioartificial membranes play critical role in medical applications such as in artificial kidneys and artificial lung, but interestingly, quite few in artificial liver.

Generally, a hollow fiber cylinder, instead of a flat porous membrane, configuration is used for BAL systems to acquire the high surface area of medium exchange and to enhance the performance efficacy in clinical tests [3]. One issue is the mechanical support of these cylinders under continuous flow, which limits the engineered design of liver bioreactor as their mechanical strength is pre-requisite for long-term perfusion [4–7]. Obviously, the porous membranes are not only readily immobilized onto rigid substrate for providing sufficient mechanical support, but they are also available commercially and easy-to-use. Another issue is to maintain the essential functions of hepatocytes onto various fibers or membranes. Although fiber-based liver bioreactors seem to have the limited success in supporting such the essential hepatic functions as protein synthesis and membrane-based liver bioreactors comprising viable hepatocytes on rigid mechanical support could be an alternative option to maintain these essential functions as, at least, physiologically relevant blood or medium flow is easily optimized by excluding the potential risk of hollow cylinder collapse.

To develop a functional liver bioreactor, a biocompatible and friendly interface between hepatocytes and substrate is crucial in providing mechanical support and maintaining cell functions. Appropriate selection of substrate membrane defines the conditioned microenvironment and procedure to permit cells to accomplish their functions. To date, few studies are reported how substrate membrane modulates the self-renewal, fate of cells and their biocompatibility because the biological features and underlying mechanisms behind biomechanical regulation are poorly understood. In this work, we systematically compared the biomechanical and biochemical characteristics of eight types of commercially available porous membranes, attempting to optimize these membranes by improving efficiency and reducing cost for future development of the potential novel bioartificial liver bioreactors. Differential membrane structure, cells adhesion, morphology and featured biomarker expressions were discussed.

## MATERIAL AND METHODS

### SEM imaging

The front or reverse side of each membrane or PS substrate was fixed on a specimen holder separately. After being electrically conducted by coating gold particles, surface images were taken using a scanning electron microscope (SEM) (Nova 200 NanoLab scanning electron microscope, USA).

### AFM compression test

Atomic force microscopy (AFM) was employed to determine the surface topography and the compression modulus of each membrane or PS substrate. Each specimen was cut from its bulk material and deposited onto freshly clean plate to fit the geometry for AFM test. AFM cantilevers (Nanosensors TM, Neuchatel, Switzerland) were applied in a contact mode to collect both the images of surface height and the force-extension curves of indentationin, a range of 512 × 512 pixels simultaneously. Off-line software NanoScope V5.30r3.sr3 (Veeco Metrology, USA) was used to reconstruct the three-dimensional surface topography profile and to determine the compression Young’s modulus, *E_c_*, using a conical model provided by manufacturers.

### Instron tensile test

All specimens of each membrane or PS substrate were cut into 10 × 1 cm in rectangle (each reserved a segment of 5 × 1 cm as the gauge section in the middle of the specimens) and tested using an Instron Micro Tester (Model 5848, USA, adopted standards: ISO527-1 and ISO527-3). Tensile Young’s modulus, *E_t_*, was calculated using $E_{t}=(\sigma_{2}-\sigma_{1})/(\varepsilon_{2}-\varepsilon_{1})$ formula where $\varepsilon_{1}=0.0005$, $\varepsilon_{2}=0.0025$ are two strain points in the tensile datum and $\sigma_{1}$, $\sigma_{2}$ are the tensile stress at the strains $\varepsilon_{1}$ and $\varepsilon_{2}$, respectively. $\sigma_{1}$, $\sigma_{2}$ Are calculated using σ_ _= *F*/*A*, $\varepsilon_{1}$, $\varepsilon_{2}$ are calculated using ε = *ΔL*/*L*, where *F* is the load, *A* is the cross-sectional area, and *L* and *ΔL* are the respective original and increment length.

### Cells and reagents

Human hepatocarcinoma HepG2 cells (ATCC, Rockville, USA) were cultured in Dulbecco’s Modified Eagle’s medium (DMEM) supplemented with 10% (v/v) fetal bovine serum (FBS; Gibco, Grand Island, USA) and 1% penicillin/streptomycin. All the cells were routinely grown at 37°C in a humidified, 95% air/5% CO_2_ atmosphere. Cells were harvested using 0.05% trypsin and 0.02% EDTA in phosphate-buffered saline (PBS, pH 7.4) when they were ∼85% confluent. Other organic reagents were purchased from Sigma-Aldrich (St. Louis, MO). Rabbit–anti-human anti-albumin (ALB) polyclonal antibodies and anti-cytokeratin 18 (CK-18) monoclonal antibodies were purchased from Abcam (Cambridge, MA). Hoechst33342 and rhodamine-labeled phalloidine from Enzo (Ann Arbor, MI) were used to stain the nuclei and actin of the cells, respectively. Bovine serum albumin (BSA) and other biochemical reagents were all purchased from Sigma-Aldrich (St. Louis, MO).

### Cell growth on membranes

Eight commercially available porous membranes were purchased from Whatman, Pall and Beijingbeihualiming, respectively (Table 1). HepG2 seeded at a density of 10^5^ cells put onto each membrane [acetyl cellulose–nitrocellulose mixed cellulose (CA–NC), CA, PTFE, JN, PP, CN, PVDF or PC] placed in a well of 12-well plastic plate (4.5 cm^2^/well) while those cells on polystyrene plates (PS; Corning, USA) in the absence of the membrane were used as control. Collagen I was pre-coated on the membrane or the PS plate in 15 µg/ml at 37°C for 2 h. The number of adhered cells on each membrane or PS substrate was visualized at 24 h after seeding by immunostaining cytoskeletal protein actin and counted using Image J software.

**Table 1.** physicochemical characteristics of biocompatible membranes

No.	Identity^a^	Color	Transmittance	Pore size (µm)	Thickness (µm)	Adhesivity	Fragility	Antistatic property	Double-sided available
1	CA–NC	White	Non-transparent	0.45	123 ± 3	+	−	−	No
2	CA	White	Non-transparent	0.45	60 ± 0	+++	+	+	Yes
3	JN	White	Non-transparent	0.45	89 ± 1	+++	−	−	No
4	PP	White	Non-transparent	0.45	182 ± 8	+++	−	−	Yes
5	NC	White	Non-transparent	0.45	148 ± 2	++	−	−	No
6	PVDF	White	Non-transparent	0.45	182 ± 8	+	−	−	Yes
7	PC	Light White	Semi-transparent	0.45	20 ± 0	+	−	−	No
8	PTFE	White	Non-transparent	0.45	140 ± 2	+++	−	−	Yes
9	PS	Colorless	Transparent		166 ± 0	++	−	−	Yes

^a^CA–NC, CA, JN, PP, NC, PVDF, PC or PTFE membrane, as well as PS slide used as control. Data are presented as mean ± SD.

### Immunological staining

Distribution of cytoskeletal proteins and biomarkers, actin, ALB or CK18, was visualized using immunostaining techniques. Cells cultured on the substrate were rinsed in PBS at pH 7.2, fixed for 30 min in 4% paraformaldehyde, and permeabilized with 0.1% Triton X-100 for 15 min. Filamentous actin was stained with rhodamine-conjugated phalloidin diluted in 1% BSA/PBS to block non-specific epitopes. Anti-ALB and anti-CK18 antibodies were added at 1:100 and 1:50 dilution in BSA/PBS, respectively. The cells were then incubated with Hoechst 33342 for 10 min at room temperature and washed twice with PBS. Collected samples were stored at 4°C followed by examination by confocal laser scanning microscopy (Zeiss L710, Germany). In some cases, actin staining was used to identify the contour of a cell for determining the projected area of the cell or the nucleus. Cell or nucleus circularity was defined as 4π × area/perimeter^2^ for quantitative comparison of morphological changes. In total, 50 cells were counted and analyzed on each membrane except of 35 cells on PC membrane.

### Protein filtration assay

Human serum was prepared from centrifugation of human whole blood of healthy donors at 3000 × *g* for 10 min after standing at room temperature for 30 min. About 1 ml of collected serum was injected onto each membrane for 30 min and the filtered medium was then collected. For SDS–PAGE analysis, equal amounts (50 µl) of the filtered medium were separated by electrophoresis on SDS–polyacrylamide gels according to standard procedures.

### Statistical analysis

Student’s two-tailed *t*-test was performed to determine the statistical significance of differences between any two parameters of nine different membranes or substrate on cell adhesion, cell and nucleus morphology, actin reorganization as well as the expression of biomarkers ALB and CK18.

## RESULTS

### Membrane structure and surface topographies

**Figure 1.**
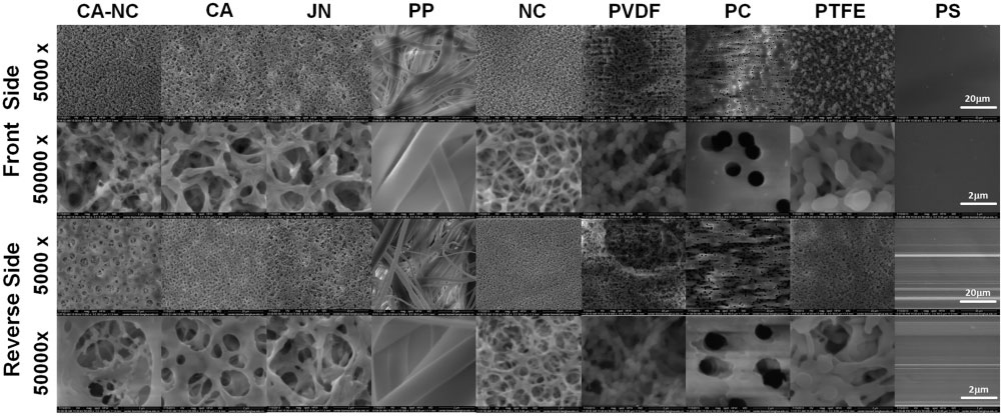
membrane structures via SEM images of eight membranes or control PS slide. Front (rows first and second) or reverse (rows third ans fourth) side of membrane or slide was viewed at low (×5000, rows first and third) or high (×50 000, rows second and fourth) magnification.

We first tested the membrane structure of the eight types of membranes. Routine eye test indicated that most of them yield the smooth surface on either single or double side(s) with one exceptional case of PP membrane where rough fibers were visualized. All the membranes appeared to be white and non-transparent except of the light white, semi-transparent PS membrane. Physicochemical features were tested and summarized in Table 1. It was found that all the membranes yield the same nominal pore size of 0.45 µm, the varied thickness of 20–182 µm, and the distinct adhesivity. Only CA membrane is fragile and antistatic. Half of the eight membranes is double-sided available for cell adhesion (CA, PP, PVDF and PTFE) but the other half is not (CA–NC, JN, NC and PC). Further SEM analysis indicated that all the membranes so tested present polyporous structures (Fig. 1), in which some pores pass through the membrane. The front or reverse side of each membrane was viewed differently. From the front view, the pores were extensive and consecutive, which makes it available for cells adhesion, growth and function maintenance due to its huge superficial area and commendable medium flow. On the reverse side, however, much fewer and discontinuous pores were seen, which seems more conducive to slow down mass transport and suitable for sufficient medium exchange.

**Figure 2.**
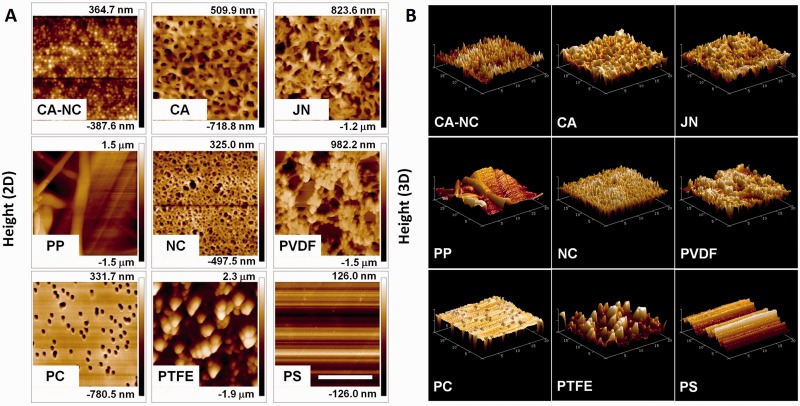
surface microtography via AFM images of eight membranes or control PS plate. Front side of membrane or plate was viewed from a 2D (A) or 3D (B) demonstration. Bar = 10 µm. Scale of height in 2D images was presented on the right scale bar of each panel in A.

We further tested the surface topography of each membrane using AFM assay. Under the same contact force, three randomly selected regimes for each membrane were scanned and the resulted surface topography was then demonstrated in two- (2D) (Fig. 2A) or three-dimensional (3D) (Fig. 2B) configuration (Fig. 2). It was found that surface topography is similar in various regimes, suggesting that all the membranes yield homogeneous structures. Typical 2D images indicated that clear, smooth pore structures (e.g*.* round holes and silky fibers) are repeated with the height of 752.3 nm to 3.0 µm along connecting pores (Fig. 2A). Such the features were confirmed using a 3D demonstration, in which the connectivity of those pore structures were more clearly visualized (Fig. 2B).

### Mechanical properties of distinct membranes

Next, we measured the mechanical properties of each membrane using indentation protocol of AFM assay and compression Young's modulus was compared among eight types of membranes. As shown in Table 2, the compression modulus was different from each other. JN, PP and PS membranes yield much higher compression modulus (5.95–6.91 MPa) than those for CA–NC, CA, NC, PVDF, PC and PTEF membranes (3.04–3.96 MPa).

**Table 2.** mechanical properties of biocompatible membranes

No.	Identity	Compression modulus (MPa)^a^ (MPa)	Tensile Modulus^b^ (MPa)	Tensile strength^b^ (MPa)
1	CA–NC	3.43 ± 0.64	102 ± 8.7	3.60 ± 0.19
2	CA	3.58 ± 0.49	565 ± 21.4	14.8 ± 0.15
3	JN	6.91 ± 3.12	707 ± 17.2	23.8 ± 0.81
4	PP	5.99 ± 2.32	143 ± 6.9	2.56 ± 0.06
5	NC	3.54 ± 1.02	397 ± 5.0	9.37 ± 0.24
6	PVDF	3.68 ± 0.78	170 ± 5.3	2.63 ± 0.12
7	PC	3.96 ± 0.58	662 ± 88.0	19.2 ± 0.75
8	PTFE	3.04 ± 1.57	188 ± 8.3	5.10 ± 0.05
9	PS	5.95 ± 0.91	3972 ± 95.2	−

^a^Measured using atomic force microscope (Vecco).^b^Measured using material testing machine (Instron). Data are presented as mean ± SD.

Tensile properties are also critical when stretching the membrane onto substrate. Thus, tensile modulus and strength were determined and compared for all the membranes. Here, four noteworthy artificial membranes of CA, JN, NC and PC yield much higher tensile modulus (397–707 MPa) than those for CA–NC, PP, PVDF and PIFE membranes (102–188 MPa), which is positively correlated to the difference in tensile strength between the former four membranes (9.27–23.8 MPa) and the latter four membranes (2.63–5.10 MPa). It was also noted that the control PS membrane yield similar compression modulus but very high tensile modulus (Table 2). Taken together, CA, JN and PP membranes tend to be potential candidates for supporting mechanically cell growth with commendable mechanical strength and surface topography. These members are ideal materials to support cell growth and are suitable to become biocompatible and friendly interface, such as BAL systems.

### Identification of hepatic cells on distinct membranes

**Figure 3.**
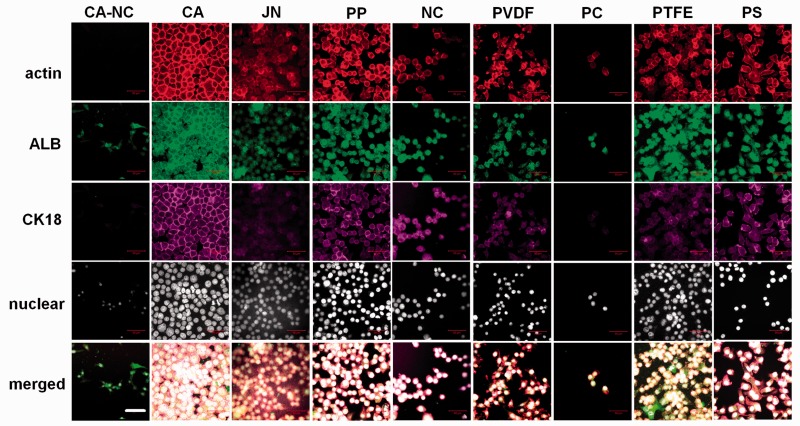
hepatic biomarker expression of HepG2 cells grown on the eight membranes or PS substrate. Data were presented as the fluorescent staining of actin (first row), ALB (second row) or CK18 (third row) proteins and of nucleus presentation (fourth row) or merged co-localization (fifth row) at *t* = 24 h at × 63 (Bar = 50 µm).

In addition to biomechanical evaluation, biological responses are also crucial when cells are seeded on the membrane. Using HepG2 cells as a cell model for hepatocytes, we also tested the cell adhesion efficacy on collagen-I pre-coated membrane. Similar to those cells on PS substrate, HepG2 cells grown on each membrane at the same seeding density were found to express two typical hepatic biomarkers of ALB (green in second row) and CK18 (purple in third row), which are co-localized with (red in first row) and merged with their nuclei (fourth and fifth rows) (Fig. 3). It was also indicated that both ALB and CK18 proteins tended to be uniformly distributed within the entire cell on each of eight membranes. These data indicated that the cells are able to grow up well and to maintain their essential functions on all the membranes. Specifically, much condenser cell population was observed on CA, JN, PP and PTFE membranes with a monolayer-like (on CA membrane) or a spheroid-like (on JN, PP or PTFE membrane) configuration. Noting that the spheroid-like configuration of hepatocytes is favorable in maintaining their functions [8, 9], these data confirmed that these membranes are friendly for the growth of hepatocytes. In contrast, relatively looser population was seen on the other four membranes with a branched configuration (first and fifth to seventh columns), suggesting that these membranes might not be favorable for cell growth. This speculation was partially confirmed by the facts that the capacity of cell adhesion is limited within 24 h and few cells were survived beyond the duration on CA–NC or PC membranes and that the number of alive cells is more and less on NC membrane as well as on PS substrate (data not shown).

**Figure 4.**
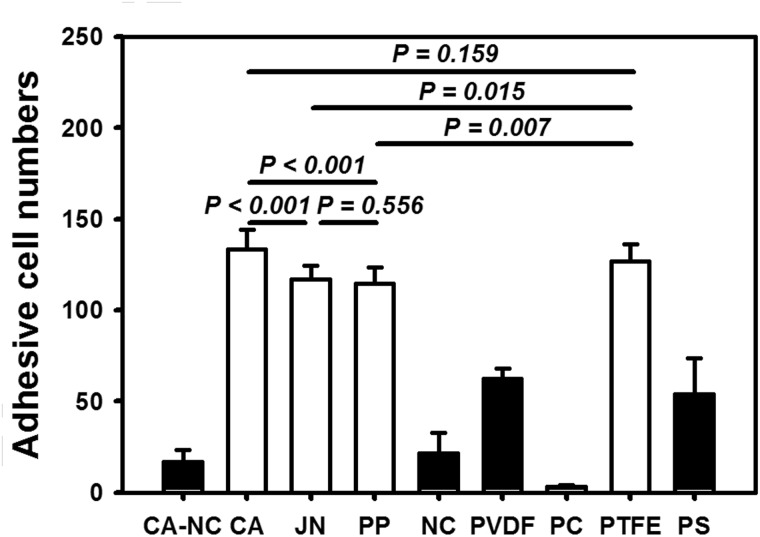
determination of adhesion capacity of HepG2 cells via number of adhered HepG2 cells on the eight membranes or PS substrate at *t* = 24 h. Totally 10 frame data were obtained from the repeats in triplets and presented as the mean ± standard deviation (SD) per frame.

To further understand the biocompatible responses of HepG2 cells on the distinct membranes, we also quantified the adhesion and spreading capacities of cells grown on each membrane as well as on PS substrate. As seen in Fig. 4, the cells adhered well at >100 cells per frame on CA, JN, PP and PTFE membrane, which is even higher than that on conventional PC substrate (54 ± 20). Among these four membranes, adhering cell number was relatively higher on CA or PTFE membrane than those on JN or PP membrane (all the values, *P* < 0.01). In contrast, cell adhesion number was lower on PVDF membrane (62 ± 6, comparable with the one on control PS substrate) or much lower on CA–NC, NC or PC membrane (from 17 ± 7, 22 ± 11, 3 ± 1). Notably, HepG2 cells were able to either possess clear morphology with well-defined boundaries or spread out with slightly less dense, smaller-sized aggregates on distinct membranes (Fig. 4), implying that the morphological analysis is also meaningful for understanding the cell behaviors on the membrane.

### Cellular morphology and spreading on distinct membranes

**Figure 5.**
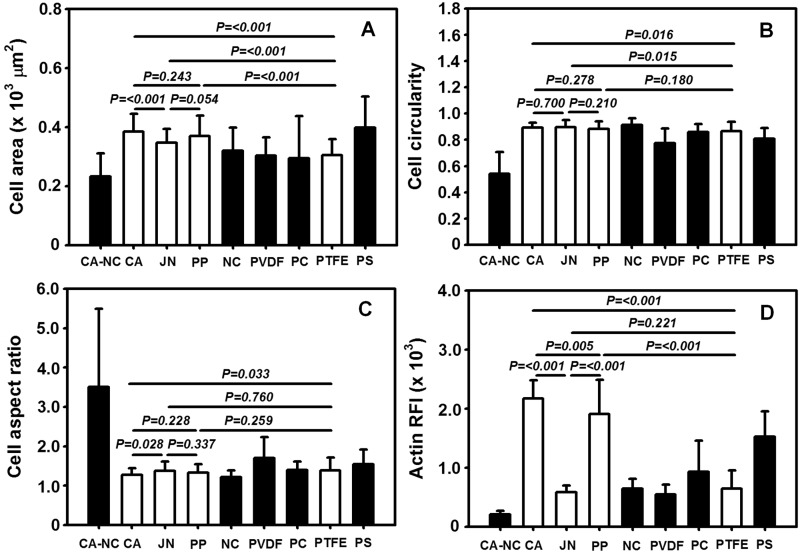
morphology and spreading of adhered HepG2 cells on eight membranes or PS substrate. Plotted are cellular projected area (A), circularity (B), aspect ratio (C) as well as relative fluorescence intensity of actin (D) at *t* = 24 h. Totally 10 frame data were obtained from the repeats in triplets and presented as the mean ± SD.

Upon the immunostaining images of actin proteins to determine the contour of a cell, the morphology of HepG2 cells was further quantified on distinct membranes. As exemplified in Fig. 5A, cell projected area was comparable on the seven of eight membranes even though slightly higher values were found on CA, JN and PP membranes (425–472 µm^2^, close to one on PS substrate at 489 µm^2^) than those on NC, PVDF, PC and PTFE membranes (360–393 µm^2^). One exceptional case came from the cells on CA–NC membrane, in which the projected area was much lower than all the other membranes (all the values, *P* < 0.01). These results suggested that the cells are favorable to grow up on CA, JN or PP membrane, at least.

Similar comparisons were performed for cellular aspect ratio, circularity and actin relative ﬂuorescence intensity (RFI). Again, no significant differences were found in cell circularity on the seven of eight membranes (0.77–0.91) except of much lower value on CA–NC membrane (0.54) (Fig. 5B). Consistent readouts were also obtained for cell aspect ratio with lower values on the seven of eight membranes (1.28–1.70) but higher value on CA–NC membrane (3.50) (Fig. 5C). Meanwhile, actin RFI value was a little diverse among the eight membranes, which reads 143% and 125% on respective CA and PP membranes, 14% on CA–NC membrane and 36–61% on other four membranes, as compared with the control value on PS substrate (Fig. 5D). Taken together, morphological, spreading and cytoskeletal analyses implied that the cells tend to favor their native morphology and spreading on CA, JN, PP or PTFE membrane, appear to work but less favorably on NC, PVDF or PC membrane, and are apt to reveal abnormal morphology and spreading on CA–NC membrane. It was also noted that both CA and PP membranes are most favorable candidates to maintain cellular functions maintain.

**Figure 6.**
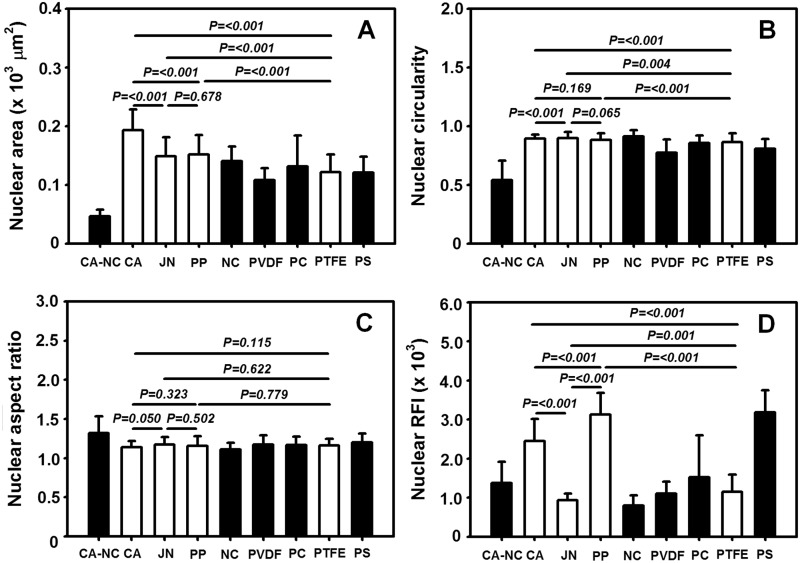
nucleus morphology and spreading of HepG2 cells on eight membranes or PS substrate. Plotted are nuclear projected area (A), circularity (B), aspect ratio (C) as well as relative fluorescence intensity of nucleus (D) at *t* = 24 h. Totally 10 frame data were obtained from the repeats in triplets and presented as the mean ± SD.

It is well known that nucleus morphology is well correlated to cellular responses on varied substrates [10]. To further address this issue, we also compared the nuclear morphology and spreading on the distinct membranes. As seen in Fig. 6, the outcomes for nuclei were in excellent agreement with those for the cells themselves, that is, nuclear projected area is higher on CA, JN or PP membrane but lower on CA–NC membrane (Fig. 6A), circularity is quite close to unity on all the membranes but slightly lower on CA–NC membrane (Fig. 6B), aspect ratio is higher on CA–NC membrane than those on the seven of eight membranes (Fig. 6C), and actin RFI value is higher on CA or PP membrane but intermediately lower on CA–NC membrane (Fig. 6D). These results supported that the cells on CA or PP membrane more likely maintain their essential functions.

### Quantification of hepatic biomarker expression and protein filtration on distinct membranes

**Figure 7.**
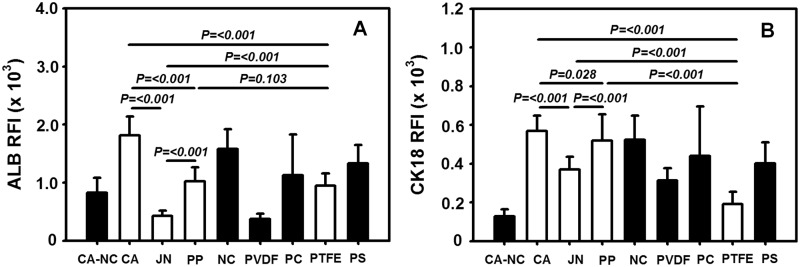
comparison of ALB (A) and CK18 (B) expressions on distinct membranes at *t* = 24 h. Data were obtained from the repeats in triplets and presented as the mean ± SD.

To further test the hepatic functions of HepG2 cells on distinct membranes, the expression of ALB and CK18 biomarkers was determined using their RFI values from immunostained images of the cells (cf. Fig. 2). It was indicated that RFI value of ALB expression is 136% and 77% on respective CA and PP membranes, as compared with the one on PS substrate. These values are higher than that on CA–NC membrane (62%), whereas they are diverse from 28% to 119% on the other five membranes (Fig. 7A). In contrast, RFI value of CK18 expression is 141% and 129% on respective CA and PP membranes, as compared with the one on PS substrate. In addition to a quite low value on CA–NC membrane (32%), the value varied from 48% to 130% on the other five membranes (Fig. 7B). These results suggested that the expression of functional biomarkers is sensitive to CA and PP membranes, which are more suitable for cell growth and functions.

**Figure 8.**
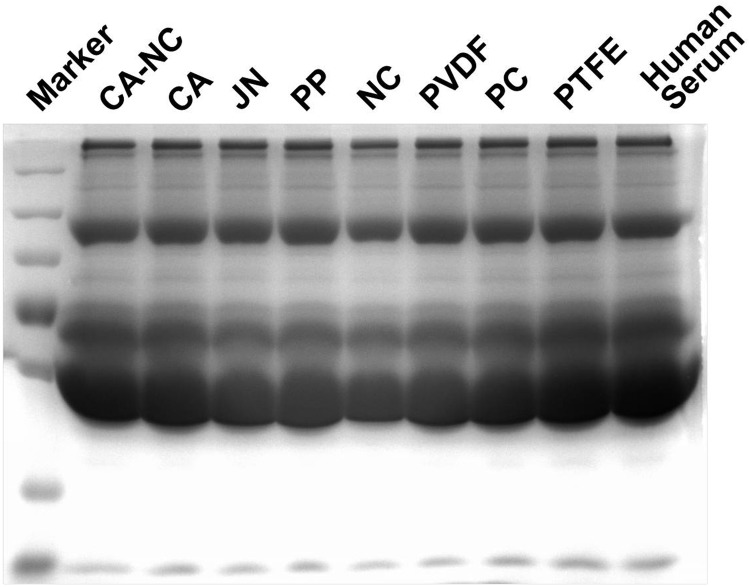
human serum filtration through distinct membranes. Filtrated components were collected and tested using SDS–PAGE electrophoresis assay.

Protein filtration across the permeable membrane is crucial for liver bioreactor. Here we employed a model protein mixture of human serum proteins to test this nature. No differences were found for protein filtration among all the membranes (Fig. 8). Noting that the mean pore size of 0.45 µm is constitutively favorable for protein filtration, these results implied that all the membranes are resistant to non-specific absorption of human serum proteins, which also supported their biocompatibility used for designing liver bioreactor.

## DISCUSSION

Most popular membrane frequently used in BAL system is hollow fiber modules with large surface area in an enclosed volume to enhance the efficacy of serum exchange. Such the BAL system and especially its core unit of liver bioreactor, however, are strictly pre-filtrated, difficult to clean and in high cost, which confines its application in regenerative medicine. Using synthetic biopolymer membranes could be another option to construct liver bioreactor because they yield well-defined surface chemistry, topography and mechanical features. The novelty of this work lies in that hepatocyte-like cells are able to maintain their essential hepatic functions on distinct bioartificial membranes, which are commercially available and easy to use. To the best of our knowledge, this is the first study to unravel systematically the respective contributions of these distinct biopolymer membranes in preserving hepatic activity, shedding light on how to replicate the *in vivo* 3D microenvironment using an optimal membrane *in vitro*.

Surface chemistry/topography and membrane mechanics are crucial in the adhesion of hepatocytes and the exchange of metabolites within liver bioreactor, especially noting that hepatocytes are hard to proliferate *in vitro*. Even with those biocompatible membranes conventionally used in life science study or in biochemical industry, they still present different capacity in their hydrophilicity or adhesivity and binding affinity for cells (Table 1). To exclude their potential effects and unify the surface chemistry, Collagen-I proteins were pre-coated on each membrane in this study by attempting to mimic the extracellular microenvironment of hepatocytes *in vivo*, as seen in liver cell biology and BAL system-based therapy [11–15]. Although those pores with a nominal size of 0.45 µm for the porous membranes are assumed to favor mass transfer and metabolite exchange between hepatocytes and plasma/blood, it should also be noted that a typical pore depicts, in reality, a random network of the unevenly shaped structures rather than a standardized cylindrical pore (Figs 1 and 2). Meanwhile, both compressive and tensile mechanics are critical for the supporting membrane as the cells are usually placed in multi-layered membranes and exposed to shear flow perfusion in a liver bioreactor. Quantifying these mechanical features provide a basis for optimizing the mechanical support for constructing the bioreactor (Table 2).

Although it is difficult to induce hepatocyte proliferation *in vitro*, long-term maintenance of hepatic becomes a key issue for applying BAL system. Bioartificial membranes are well known to regulate hepatocytes adhesion, growth and activity when an *in vitro* microenvironment matches the optimally developed *in vivo* scenario [11, 16–21]. Recently, growing evidences indicated that biological behaviors of hepatocytes are correlated to matrix topography, optimization of extracellular microenvironment and the control of cell–cell interactions in long-term culture of hepatocytes [22–25]. It was also noted that surface chemistry/topography of the membrane plays a key role in cell spreading [26–29], locomotion [30] and proliferation [31]. In this study, distinct membranes with different surface topography and mechanical feature presented diverse appearance in regulating cell and nucleus morphology, cell adhesion, actin organization and ALB and CK18 expressions (Figs 3–7). These findings are also in agreement with those in the literatures. For example, mechanical clues regulate hepatocyte functions by altering their shape and cytoskeletal network [28, 32, 33]. In addition to the substrate stiffness defining the fate of hepatocytes by altering the cellular traction force and the nuclear translocation of transcription factors, the substrate topography also regulates adhesion and proliferation by modifying the distribution of focal adhesion complexes and modulating the cell traction [34]. These physical or mechanical signals have differential effects on hepatocyte functions such as ALB and CK18 expressions [35–38].

From the viewpoint of applying BAL system-based therapy, there are a lot of clinical and pre-clinical evidences having identified the following scenarios: (i) critical mass is required by loading ∼10^10^ hepatocytes or hepatocyte-like cells into a specialized bioreactor; (ii) like-organoid aggregates in the porous matrix are preferential to make compact contact among cells for enhancing cell density and prolonging hepatic functions; (iii) features of the porous membrane govern the number and quality of cells and the exchange rate and efficiency of plasma/blood. Although the first two issues are beyond this study, appropriate bioartificial membrane should be taken as an important issue in designing a liver bioreactor. Although majority of the current BAL prototypes have adopted the configuration of hollow fiber bioreactor [11, 12, 17, 39, 40], a flat membrane bioreactor, so-called a ‘sandwich culture’ device, was recently proposed as the most stable culture method for hepatocytes in BAL system [15, 19, 20]. Our work here identified the selectivity of distinct biocompatible membranes, i.e. CA and PP membranes serving as favorable candidates for future liver bioreactor construction. More tests should be performed using various kinds of hepatocytes or hepatocyte-like cells in the future even though the modeled HepG2 cells express normal liver-specific pathways such as ureogenesis, gluconeogenesis and cytochrome P-450 activities and produce albumin and α-fetoprotein. Related downstream pathways such as integrin, RhoA and ROCK of the cells will also be clarified in the future work.

## CONCLUSION

Although the interactions between the porous membrane and HepG2 cells were determined to mimic cellular responses in liver, the sensitivity and capacity of the membranes to cell functions are dramatically different in their topographical and mechanical aspects. The mechanisms underlying the differences involve their differential abilities to alter cellular morphology and functions independently. These results imply the differential predominance of mechano-biological responses for these bioartificial membranes, particularly when they are considered to be a therapeutic source for hepatic regenerative applications. Therefore, our results provide useful information that may assist the development of next-generation BAL systems.

## FUNDING

This work was supported by Strategic Priority Research Program of Chinese Academy of Sciences (grant XDA01030604); National High Technology Research and Development Program of China (grant 2011AA020109); National Natural Science Foundation of China (grants 31110103918 and 31470907); National Key Basic Research Foundation of China (grant 2011CB710904).

*Conflict of interest statement*. None declared.
